# Maternal and fetal outcome in placenta accreta spectrum (PAS) associated with placenta previa: a retrospective analysis from a tertiary center

**DOI:** 10.25122/jml-2021-0134

**Published:** 2021

**Authors:** Valentin Nicolae Varlas, Roxana Georgiana Bors, Simona Birsanu, Bogdan Maxim, Eliza Clotea, Maria Mihailov

**Affiliations:** 1. Department of Obstetrics and Gynaecology, Filantropia Clinical Hospital, Bucharest, Romania; 2. Department of Obstetrics and Gynaecology, Carol Davila University of Medicine and Pharmacy, Bucharest, Romania

**Keywords:** placenta accreta spectrum, placenta previa, accreta, increta, percreta, maternal and fetal outcome

## Abstract

Accreta placenta spectrum is a complex obstetrical condition of abnormal placental invasion associated with severe maternal morbidity. This study aimed to analyze our therapeutic management and counseling of the cases with placenta accreta spectrum (PAS) associated with placenta previa. We performed a retrospective study of pregnant women with PAS associated with placenta previa at the Filantropia Clinical Hospital between January 2017–April 2021. In these cases, the earlier diagnosis was realized by an ultrasonographic scan and was confirmed by histopathological findings after the surgical treatment. The conservative management was obtained in one case at <37 weeks of gestation, and the maternal outcome was uterine preservation. Among the 12 patients, the mean age was 34±3.44 years. All women had risk factors for abnormally invasive placenta, such as placenta previa or previous cesarean delivery. Most women underwent planned cesarean delivery at the mean gestational age of 36.4±0.9 weeks. In our study, the uterus was preserved in only one case (8.33%), and hysterectomy with preservation of ovaries was performed in the rest of the cases. Mean maternal blood loss during surgery was 2175±1440 ml. Severe maternal outcomes were recorded only in one case (8.33%). We identified a low uterine preservation rate and a good perinatal outcome. Conservative management should be reserved for fertility desire and extensive disease due to surgical difficulty. Early identification of the risk factors and strategic management may improve maternal and fetal outcomes.

## INTRODUCTION

**Figure 1:**
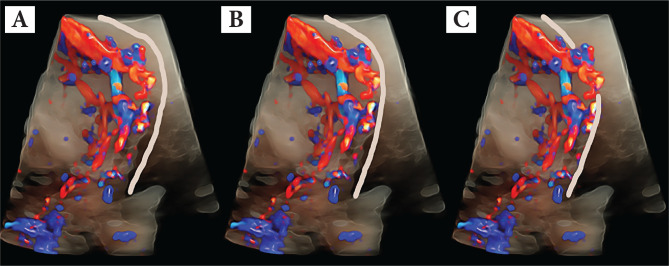
Placenta accreta spectrum (PAS), with all grades of abnormal placentation (A-accreta, B-increta and C-percreta).

Placenta accreta spectrum (PAS) is a term used to describe abnormal placental invasion, including placenta accreta (penetration <50% of the myometrium), increta (penetration >50% of the myometrium) and percreta (penetrates beyond the myometrium to the uterine serosa and adjacent organs) [1]. The principal societies – International Federation of Gynaecology and Obstetrics (FIGO) [2], Royal College of Obstetricians and Gynaecologists (RCOG) [3] and the American College of Obstetricians and Gynecologists (ACOG) [4] – defined the term placenta accreta spectrum (PAS), with all grades of abnormal placentation (Figure 1).

It is a consequence of a defective decidualization determined by placental implantation at an area of preexisting damage to the endometrial-myometrial interface [5]. Previous uterine surgery such as cesarean section, hysteroscopic removal of intrauterine adhesions, cornual resection of ectopic pregnancy is most frequently associated with PAS. Other factors that may be involved in the development of PAS are previous curettage, submucous fibroids, or uterine malformations [6].

The most important risk factor for the development of a PAS is placenta previa after a prior cesarean delivery/section. The difficult diagnosis of this pathology explains the variation in placenta accreta prevalence between 1 in 300 and 1 in 2000 pregnancies [3]. In the case of women with a prior single cesarean delivery, the presence of placenta previa is associated with a 3% risk of PAS, while the absence of placenta previa is associated with a 0.03% risk of PAS. In a recent meta-analysis, Jauniaux *et al*. indicate a PAS prevalence between 0.01% and 1% [7]. This difference of risk is even more evident in women with a history of multiple cesarean sections. Women over 35 years or with a personal history of pelvic irradiation, manual removal of the placenta, endometritis, or infertility have a higher PAS risk compared to control groups [8].

The increased rate of cesarean sections led to a higher prevalence of PAS. Progress was made both in the detection and treatment of these cases. Most patients with PAS can be detected prenatally, and appropriate management can be planned. The cesarean delivery should take place in a tertiary center with the participation of a multidisciplinary team that includes a neonatologist, obstetrician, urologist and anesthesiologist [1].

## MATERIAL AND METHODS

**Figure 2:**
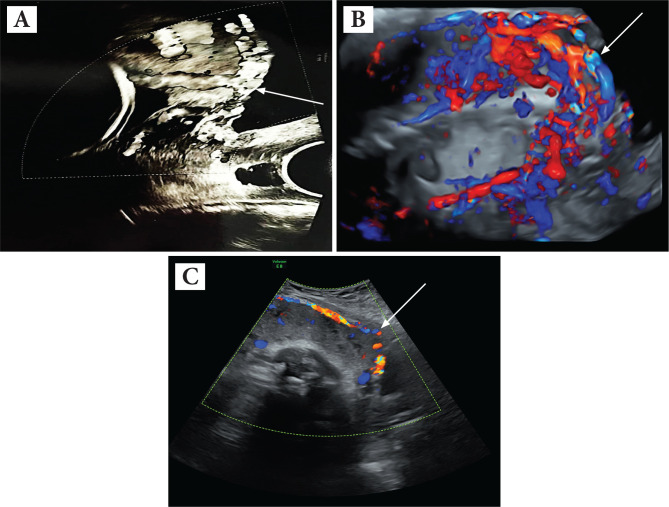
Ultrasound findings: (A) anterior placenta previa with abnormal lacunae, neovascularity, (B) bulge sign, no myometrium detectable, no clear zone, hypervascularity; (C) neovascularity with abnormal lacunae.

We performed a retrospective study of 12 pregnant women with PAS and placenta previa identified and treated at the Filantropia Clinical Hospital, Bucharest, from January 2017 to April 2021. The diagnosis was suspected after the ultrasonographic scan and was confirmed after surgery by the intraoperative findings and by the pathological report. In some cases where bladder invasion was suspected, magnetic resonance imaging (MRI) was used to assess the degree of bladder involvement. The ultrasound evaluation of the interface between the placenta and myometrium was done at a gestational age between 18 and 24 weeks. As a result, an early diagnosis of suspicion was made in 75% of cases (Figure 2).

In 2 (16.6%) cases, the suspicion diagnosis was made in the third trimester when the patients presented to the hospital with symptoms such as bleeding, uterine contractions, or reduced fetal movement. In one (8.3%) case, the diagnosis of PAS was made during the cesare-an section for placenta previa. The patients with placenta previa associated with PAS were divided into two groups according to the PAS risk factors: group A (83.33%) had only one prior cesarean delivery, and group B (16.67%) had two cesarean deliveries. We compared the fetal and maternal outcomes after the surgical intervention.

## RESULTS

Among the 12 patients, the mean age was 34±3.44 years. The analysis of the risk factors for placenta previa associated with PAS shows that the most important risk factor for PAS is the presence of a prior cesarean section (all cases). The risk for PAS is higher in the group of patients with multiple uterine surgical interventions (16.6% of cases) compared with those with only one prior cesarean section (83.3% of cases). The demographic and obstetric characteristics collected from the observation sheet of the patient were analyzed using Excel 2017 for Windows and are presented below (Table 1).

**Table 1:** Demographic and obstetric characteristics of the patients and neonates.

Demographic data	High-risk pregnant women with placenta previa and PAS (n=12)
**Maternal age, mean (SD) years**	34±3.4
**Area of residence**	
Urban (n)	9 (75%)
Rural (n)	3 (25%)
**Parity**	
Primiparous (n)	0
Multiparous (n)	12 (100%)
**Patients with previous cesarean section**	
1	10 (83.3%)
2	2 (16.7%)
**Gestational age**	35.8 ± 2.3
**Placenta previa topography**	
Anterior	9 (75%)
Posterior	3 (25%)
**Blood loss, mean (SD)**	
500–1000 ml	3 (25%)
1000–1500 ml	1 (8.3%)
1500–2000 ml	3 (25%)
2000–2500 ml	1 (8.33%)
>2500 ml	4 (33.3%)
**Neonatal outcomes**	
Birth weight (grams), mean (SD)	2696.3±466.3
NICU admission, n (%)	1 (8.33%)

NICU – neonatal intensive care unit.

Most women (91.6%) underwent planned cesarean delivery at the mean gestational age of 36.4±0.9 weeks. In only one case, the patient underwent an emergency cesarean section for intense abdominal pain and severe bleeding. Fetal Apgar scores were not significantly different in the group of patients who underwent planned cesarean section and did not depend on the risk factors for PAS (the mean fetal Apgar score was 9). However, the patient with emergency cesarean section had a lower fetal Apgar score, namely 7. This fact shows the importance of an early diagnosis and a close follow-up of the patients with this pathology. Fetal Apgar scores were not significantly affected by the depth of placental invasion but were influenced by the gestational age at the moment of delivery.

In our study, the uterus was preserved in only one case (8.3%). In this case, the pregnancy was full term (37 weeks of gestation), fetal Apgar score was 9 at 1 minute after birth and 10 at 5 minutes after birth. The patient had a history of one prior cesarean section and was suspected of having placenta accreta in the current pregnancy. A fundal uterine incision was made, extended to the posterior uterine wall of approximately 8–10 cm in length. No placental separation signs were observed after infant delivery. There was no active bleeding, and the decision to leave the placenta in situ was made. Blood loss was estimated to be around 500 mL. The complete resorption of the placenta occurred in the following months with no significant complications associated.

Conservative management by abandoning the placenta in situ was also tried in the case of a placenta accreta at a pregnancy of 37 weeks. However, an important postpartum hemorrhage 4 days after the cesarean section imposed an emergency hysterectomy with left ovary removal.

**Figure 3:**
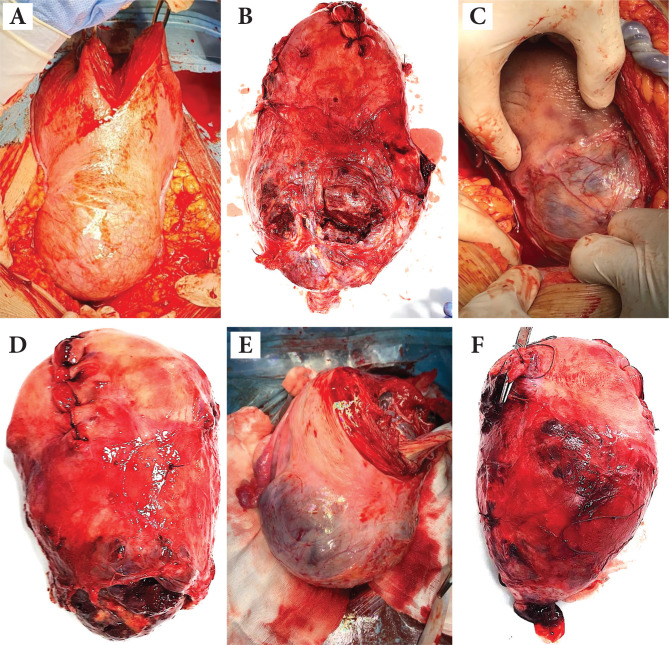
Different uterus specimens after hysterectomy: (A) placenta accreta, (B) placenta percreta with bulge, (C) placental bulge, neovascularity, invasive increta/percreta, (D) anterior placenta previa with placenta increta, (E) placental bulge under intact serosa, neovascularity, (F) placenta increta, with neovascularity and no macroscopic invasion into uterine serosa.

**Figure 4:**
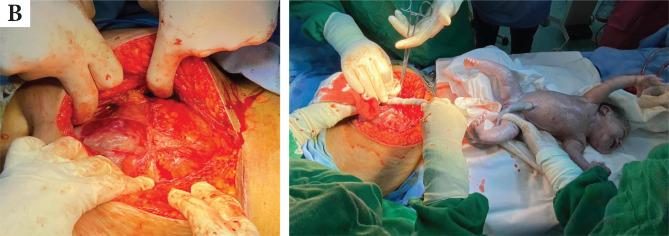
(A) intraoperative findings of placenta increta; (B) extraction of the newborn by high corporeal incision of the uterus.

In the great majority of cases (83.4%), a cesarean hysterectomy with preservation of ovaries was performed (Figures 3, 4). The mean maternal blood loss during surgical treatment was 2175±1440 ml.

In the emergency cesarean section case, the patient presented at the hospital for intense abdominal pain, which did not subside under tocolysis and analgesia. The patient had a history of prior cesarean section. The gestational age of the current pregnancy was 29 weeks and one day. The patient was taken up for emergency laparotomy, and a hemoperitoneum of 1000 ml was found along with a uterine midline and inferior defect, where the placenta was emerging. The fetus was extracted, weighing 1500 g and receiving an Apgar score of 6 at 1 minute after birth and 7 at 5 minutes. Bladder invasion was also documented. Suture of the uterus was attempted but could not be performed due to the fragile uterine wall penetrated by the placenta. A total hysterectomy was performed to stop the massive hemorrhage. When separation of the bladder from the uterine wall was attempted, bladder injury occurred and cystorrhaphy had to be carried out under difficult circumstances. Due to the massive hemorrhage (5500 ml), the patient suffered a hemorrhagic shock. She was transfused several times intraoperatively and postoperatively. She was intensively monitored postoperatively and was transferred on the second postoperative day to a multidisciplinary hospital for hemodialysis and observation.

Blood loss differed significantly in the group with only one prior cesarean section (1800±1300 ml) compared with the group with multiple cesarean sections (3400±100 ml). However, intraoperative blood loss was higher in the case of non-conservative management (2000±1400 ml) compared with conservative surgery (500 ml). Bladder injury was reported in 3 out of 12 cases (25%), only in cases of non-conservative surgical treatment. Bladder invasion was suspected preoperatively and documented intraoperatively in one case (8.3%).

Short-term complications were reported more often in the group of patients with conservative management compared to those who underwent hysterectomy and included delayed hemorrhage. No severe maternal outcome was reported after conservative management of placenta previa associated with PAS.

## DISCUSSION

The highest incidence of PAS cases is in the lower uterine wall in the area of the post-cesarean scar, involving the cervix in cases of coexisting placenta previa.

The increase in cesarean rates in most middle and high-income countries led to an increase in the prevalence of PAS. Following a single cesarean, the risk of placenta previa is 50% higher. Studies suggest that elective cesarean deliveries may be associated with a higher PAS risk compared to emergent cesarean deliveries [3, 9]. Existing data show that a prior myomectomy is associated with a very low risk of PAS. Possible factors that may induce a false conclusion include the surgical techniques used for cesarean delivery and myomectomy. For example, myomectomy with entry into the uterine cavity and a large myometrial scar may influence the risk of PAS [10].

The surgical technique used for closing the uterus during cesarean delivery could play a role in the etiology of PAS. Studies suggest that a single-layer uterine closure compared with a multiple-layer uterine closure, locked versus interrupted suturing or suture materials could influence the risk of PAS in future pregnancies [11]. However, single-layer closure of the uterine incision is associated with a reduction in mean blood loss and duration of the operative procedure [12]. More studies are necessary to assess the impact of surgical techniques used during cesarean delivery on the risks of PAS [10].

Ultrasound can assess the topography of the placental invasion, the degree of vascularization in the lower uterine segment and the depth of the area of abnormal adhesion, as well as the invasion of other structures determining factors of maternal morbidity [13].

In the second and third trimesters of pregnancy, the following ultrasound markers have been associated with PAS: placental lacunae (large, irregular intraplacental sonolucent spaces in the center of a cotyledon) with high-velocity feeder vessels, disruption of the bladder wall-uterine serosa interface (the “bladder line”), disruption of the normal hypoechoic area behind the placenta (known as the “clear space”), abnormal vascularity (vessels that extend from the placenta through the myometrium into the bladder or the serosa), placental bulge, and a focal exophytic mass (most often seen inside a filled urinary bladder) [14, 15]. The “clear space” can be obscured at an advanced gestational age by pressure from the ultrasound probe and bladder filling or by posterior placental location. Color Doppler is helpful in conjunction with the other ultrasound findings. Doppler markers of PAS include diffuse or focal placental lacunar flow, vascular lakes with turbulent flow, and hypervascularity of the serosa-bladder interface [16].

The accuracy of first-trimester ultrasound evaluation for the diagnosis of PAS is less documented. PAS should be suspected if ultra-sound examination during the first trimester reveals a gestational sac in the lower uterine segment in the proximity of a uterine scar or a very thin (<5 mm) anterior myometrial thickness. Other findings such as intraplacental sonolucent spaces and the disruption of the bladder line may also be observed [17, 18]. The bladder wall-uterine serosa interface is better visualized during ultrasound examination with a full bladder (approximately 200–300 mL) [8]. The positive predictive value of ultrasonography for placenta accreta ranges from 65% to 93%. This is why it is recommended to wait for spontaneous placental separation after delivery of the infant during the surgical intervention. The absence of placenta separation confirms placenta accreta clinically [4]. The use of Doppler ultrasound markers has increased the sensitivity of ultrasound examination to around 90%. The prenatal detection rates depend on the ultrasound signs used, operator's experience, scanning conditions, equipment used, and gestational age [7].

**Figure 5:**
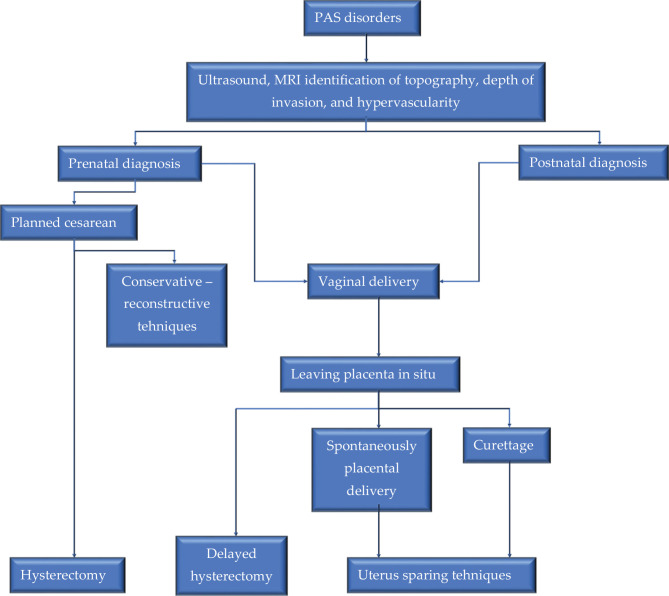
Flow diagram of PAS management with or without placenta previa.

The solution of these cases must be done in a tertiary center with interventional experience, with an intensive care unit for premature babies, and possibly with interventional radiology. In practice, we frequently encounter two scenarios; first, the case of a patient that is hemodynamically stable without bleeding episodes, in which case a solution plan can be established in case of emergency and second, the case of an unstable patient with different degrees of bleeding up to hemorrhagic shock, a situation in which the patient's management plan will be individualized and made ad hoc depending on the location and degree of placental invasion (Figure 5).

Recommendations for treating placenta previa associated with PAS vary widely and are primarily based on case series and reports, personal experience, expert opinion, and clinical judgment. All women in our study received antenatal corticosteroids between 23 and 34 weeks of gestation. Delivery was planned at 37 weeks of gestation with the availability of blood products and a multidisciplinary team was ensured [2]. However, hysterectomy of necessity following a planned cesarean section at 34–35 weeks with placental abandonment in situ is the ACOG-recommended procedure for PAS [19].

Management of the placenta with abnormal adherence is mainly radical by performing a peripartum hysterectomy. In recent years, taking into account the increasing incidence of this condition secondary to the increase in the number of cesarean sections, studies have led to the identification of conservative techniques, such as intentional placental abruption, partial myometrial excision and the “Triple P procedure”. “The triple P procedure” includes the following steps: preoperative placental topography to identify the upper edge of the placenta; use of occlusal balloons in the iliac arteries to reduce blood flow to the placental bed and resection of the placenta with the underlying adherent myometrium without separation, followed by suture of the myometrium [20].

The recommended management of placenta accreta is planned preterm cesarean hysterectomy with the placenta left in situ [21]. The removal of the placenta is associated with significant hemorrhagic risk. Extraction of the newborn, unaccompanied by the placenta separation, requires leaving the placenta in place, hysterorrhaphy, and performing a hysterectomy, with a low risk of bleeding. There are situations during hysterectomies in which a deliberate cystotomy can be considered to separate the placental tissue from the bladder.

The RCOG and ACOG protocols recommend judicious transfusion monitoring of blood loss, correction of coagulation disorders, as well as hydroelectrolytic disorders. Follow-up of the patient postoperatively in the intensive care unit continues the correction of vital parameters.

However, there are many women who want to maintain fertility. In these cases, this approach may not be the first-line treatment. Thus, conservative or expectant management can be considered. In the case of reduced areas of abnormal adhesion, the uteroplacental tissue can be resected using the uterus-sparing surgical technique. Another procedure involves the use of the Bakri balloon [17]. In the case of a large area of abnormal adhesion, the placenta is usually left in situ. Therefore, the therapeutic decision should be individualized [4].

The prognosis is better in patients who have placenta accreta without a placenta previa due to a higher risk of bleeding and hysterectomy, respectively. An additional risk is presented by patients with the placenta percreta, which may be associated with an increased incidence of urological lesions, and sometimes massive blood loss.

In a multicentre retrospective case series on 452 patients on the management of invasive placenta, Palacios *et al*. showed that when using the resection-reconstruction approach (one-step conservative surgery), the uterus can be preserved with minimal morbidity and blood loss in almost 80% of cases [22].

Subsequent pregnancies after conservative technique (resection-reconstruction) for PAS have shown similar perinatal outcomes to cesarean delivery, with no differences regarding the pregnancy course [23].

The main risk associated with PAS is severe obstetric hemorrhage, which causes secondary complications including coagulopathy, multisystem organ failure, and death. Surgical risks increase with the depth of placental invasion. Studies report the following incidence of surgical complications in women with PAS – bladder injury: 5–40%, ureteral injury: 0–18%, bowel injury/obstruction: 2–4%, venous thromboembolism: 4%, surgical site infection: 18–32%, maternal mortality: 1–7%, large-volume blood transfusions: 5–40% [24]. Many studies have evaluated the role of prophylactic placement of balloon occlusion catheters to reduce bleeding at the time of cesarean hysterectomy for PAS. However, there is insufficient evidence to demonstrate the real safety and efficacy of these devices and establish which groups of patients with PAS would have more benefit from their use. Other studies analyzed the advantage of ligating the internal iliac arteries and concluded that the efficacy is similar to balloon occlusion devices [25, 26].

When placenta previa associated with PAS is suspected, surgeons should consider uterine incision at a site distant from the placenta and should deliver the baby without disturbing the placenta in order to enable conservative management or elective hysterectomy with a reduced blood loss [3, 4]. The abdominal incision must allow sufficient access to the uterus, and hysterotomy should be above the upper placental margin. Preoperative or intraoperative ultrasound examination enables a good visualization of the upper placental margin, and as a result, helps the surgeons to establish both the abdominal and uterine incision [8]. The choice of skin and uterine incisions required to avoid the placenta depends on the placenta's location. A Pfannenstiel skin incision allows good visualization of the lower uterine segment and is recommended if the upper margin of the anterior aspect of the placenta does not rise above the lower segment of the uterus. When the placenta extends above the lower uterine segment, a midline skin incision is needed to allow a high longitudinal uterine incision.

Women who are candidates for conservative management should be warned of the risks of bleeding and infection. Studies failed to confirm the benefit of methotrexate or arterial embolization to reduce these risks, and as a result, these treatments are not recommended by the current guidelines [3, 4]. A progressive decrease in blood circulation within the uterus and the placenta with necrosis of the villous tissue and a progressive detachment of the placenta from the uterus is expected [3].

There is insufficient evidence to recommend magnetic resonance imaging and/or serial evaluation of serum beta-human chorionic gonadotropin (β-hCG) to monitor cases that were managed conservatively. Patients should undergo weekly follow-up visits during the first two months and then monthly visits until complete resorption of the placenta. The follow-up should include a clinical examination (bleeding, temperature, pelvic pain), ultrasound (size of retained tissue), and laboratory tests (hemoglobin and leukocytes count, vaginal sample for bacteriological analysis). Postoperative antibiotic therapy (amoxicillin and clavulanic acid) should be administered to reduce the risk of infection. Subsequent pregnancies are at increased risk for recurrent PAS, uterine rupture, postpartum hemorrhage, and peripartum hysterectomy. The average risk of recurrence of PAS is approximately 22–29%, and the risk of early postpartum hemorrhage is approximately 8.6–19%. Long-term complications include intrauterine adhesions and secondary amenorrhea with secondary infertility [2].

## CONCLUSIONS

Prenatal diagnosis and leaving the placenta in situ may be associated with reduced maternal morbidity. In this retrospective study, we identified a low successful uterine preservation rate, a low maternal complication rate, and a relatively good fetal outcome. After very careful prenatal counseling, we recommend that hysterectomy should be used as the treatment of choice for morbidly adherent placenta associated with placenta previa. Conservative management should be reserved for women with a strong fertility desire and women with an extensive disease that precludes primary hysterectomy due to surgical difficulty. Early identification of the risk factors and strategic management may improve maternal and fetal outcomes.
